# Study of expression analysis of *SIRT4* and the coordinate regulation of bovine adipocyte differentiation by *SIRT4* and its transcription factors

**DOI:** 10.1042/BSR20181705

**Published:** 2018-12-14

**Authors:** Jieyun Hong, Shijun Li, Xiaoyu Wang, Chugang Mei, Linsen Zan

**Affiliations:** 1 College of Animal Science and Technology, Northwest A&F University, Yangling 712100, Shaanxi, China; 2 National Beef Cattle Improvement Center, Northwest A&F University, Yangling 712100, Shaanxi, China

**Keywords:** adipocyte, bovine, promoter region, SIRT4, transcription factor, transcription regulation

## Abstract

Sirtuins, NAD^+^-dependent deacylases and ADP-ribosyltransferases, are critical regulators of metabolism involved in many biological processes, and are involved in mediating adaptive responses to the cellular environment. SIRT4 is a mitochondrial sirtuin and has been shown to play a critical role in maintaining insulin secretion and glucose homeostasis. As a regulator of lipid homeostasis, SIRT4 can repress fatty acid oxidation and promote lipid anabolism in nutrient-replete conditions. Using real-time quantitative PCR (qPCR) to explore the molecular mechanisms of transcriptional regulation of bovine *SIRT4* during adipocyte differentiation, we found that bovine *SIRT4* is expressed at high levels in bovine subcutaneous adipose tissue. *SIRT4* knockdown led to decreased expression of adipogenic differentiation marker genes during adipocyte differentiation. The core promoter of bovine *SIRT4* was identified in the −402/−60 bp region of the cloned 2-kb fragment containing the 5′-regulatory region. Binding sites were identified in this region for E2F transcription factor-1 (E2F1), CCAAT/enhancer-binding protein β (CEBPβ), homeobox A5 (HOXA5), interferon regulatory factor 4 (IRF4), paired box 4 (PAX4), and cAMP responsive element-binding protein 1 (CREB1) by using Electrophoretic mobility shift assay (EMSA) and luciferase reporter gene assay. We also found that E2F1, CEBPβ, and HOXA5 transcriptionally activate *SIRT4* expression, whereas, IRF4, PAX4, and CREB1 transcriptionally repress *SIRT4* expression. We further verified that *SIRT4* knockdown could affect the ability of these transcription factors (TFs) to regulate the differentiation of bovine adipocytes. In conclusion, our results shed light on the mechanisms underlying the transcriptional regulation of *SIRT4* expression in bovine adipocytes.

## Introduction

Sirtuins are a conserved family of proteins comprising NAD^+^-dependent deacetylases and ADP-ribosyltransferases, and thus their functions are intrinsically connected with cellular metabolism [1]. Accumulating evidence has indicated that sirtuins are important sensors of energy status and that they protect cells against metabolic stress [2]. The biological regulatory functions of sirtuins vary from metabolism to cell survival, and sirtuins are involved in a range of physiological and pathological activities, such as control of ageing [3,4], longevity pathways [5], DNA repair [6], transcriptional silencing [3], apoptosis [7], and the control of metabolic enzymes [8]. Sirtuins regulate these processes and are in turn regulated by diet and environmental stress. The mammalian sirtuin family contains seven members (SIRT1–7), which function to regulate metabolism in a non-redundant manner in many tissues [9]. Because each member exhibits distinct targets, functions, and subcellular localizations such as the nucleus (SIRT1, SIRT2, SIRT3, SIRT6, SIRT7), cytoplasm (SIRT1, SIRT2), and mitochondria (SIRT3, SIRT4, SIRT5), sirtuins can coordinate cellular responses to calorie restriction throughout the organism [10,11].

SIRT4 is a member of mitochondrial sirtuins, which, because of their dependence on NAD^+^ as a cofactor, are metabolic sensors of cellular energy status [12]. Although it was originally reported to be an ADP-ribosyltransferase that is located in the mitochondrial matrix, SIRT4 is capable of removing several acyl-groups from lysine residues. Previous studies have reported that SIRT4 affects important biological pathways, especially those involved in metabolic diseases such as diabetes, obesity, and cancer [13–16].

*SIRT4* is highly expressed in islet β cells and can interact with adenine nucleotide translocator 2/3 (ANT2/3) and insulin degrading enzyme (IDE) [17]. SIRT4, through its ADP-ribosylation action, down-regulates the enzymatic activity of glutamate dehydrogenase (GDH) an enzyme, which promotes the metabolism of glutamate and glutamine, generates ATP, and stimulates insulin secretion. Therefore, SIRT4 represses the ability of β cells to secrete insulin in response to these amino acids [18]. On the other hand, SIRT4 removes three acyl moieties from lysine residues, namely methylglutaryl (MG)-, hydroxymethylglutaryl (HMG)-, and 3-methylglutaconyl (MGc)-lysine. The metabolites leading to these post-translational modifications are intermediates in leucine oxidation, and SIRT4 plays a key role in controlling this pathway [19]. Leucine has been identified as a potent insulin secretagogue in pancreatic islets [20], and studies have shown that dysregulated leucine metabolism in *SIRT4* KO mice leads to elevated basal and stimulated insulin secretion, which progressively develops into glucose intolerance and insulin resistance [21]. These findings identify a robust enzymatic activity for SIRT4, and suggest it as a crucial player in maintaining insulin secretion and glucose homeostasis.

SIRT4 acts as a key factor of adipogenesis, regulating the proliferation and differentiation of preadipocytes [22,23]. Recent studies have identified that SIRT4 acts as a critical regulator of lipid homeostasis [24], inhibits fatty acid oxidation, and promotes lipid anabolism, which indicates that SIRT4 can regulate the balance between fat oxidation and synthesis. SIRT4 also regulates lipid homeostasis by deacetylating malonyl-CoA decarboxylase (MCD). By inhibiting MCD via deacetylation and resulting in a buildup of malonyl-CoA, SIRT4 reduces carnitine palmitoyltransferase-1 (CPT1) and subsequently blocks fatty acid oxidation. SIRT4 not only inhibits fatty acid oxidation (in skeletal muscle), but also induces lipogenesis in white fatty tissue through regulation of MCD activity [25]. Moreover, *SIRT4* KO mice showed increased fatty acid oxidation in various tissues such as the liver and myotubes, and were protected from obesity caused by high-fat diet, while remaining insulin resistant. Therefore, we can infer that SIRT4 regulates insulin sensitivity via energy metabolism in numerous metabolic tissues [22]. However, the molecular mechanisms associated with bovine *SIRT4* expression during adipose development are still unclear.

Transcriptional regulation is a vital physiological process in all living organisms. It is orchestrated by transcription factors (TFs) and regulatory proteins working coordinately together [26]. Transcriptional regulation allows the cell or organism to respond to a variety of intracellular and extracellular signals and thus adapt accordingly. Promoter is a region of DNA fragment, which may bind RNA polymerase, TFs, and other proteins for the successful initiation of transcription directly upstream of the particular gene [27]. In general, the promoter is located near the transcription start site (TSS) of the gene, and upstream on the DNA toward the 5′ region of the sense strand, and TFs can be divided in two main categories, namely activators and repressors [28]. The regulation of sequence-specific TFs such as E2F TF-1 (E2F1), CCAAT/enhancer-binding protein β (CEBPβ), homeobox A5 (HOXA5), interferon regulatory factor 4 (IRF4), paired box (PAX) 4 (PAX4), and cAMP responsive element-binding protein 1 (CREB1) is coordinated in part by the action of adipocytes [29–34]. However, the relationship between differentiation and the transcriptional regulation of the *SIRT4* promoter activity still needs to be clarified.

In the present study, the mechanisms underlying the molecular regulation of the *SIRT4* promoter were analyzed. In addition, we determined the relative mRNA expression pattern of bovine *SIRT4* in tissues, and its subcellular localization in bovine adipocytes. Thus, the present study aimed to elucidate the transcriptional regulation of *SIRT4* in bovine adipocyte differentiation.

## Materials and methods

### Ethics statement

All the experimental procedures on cattle used in the present study have been approved by the Experimental Animal Manage Committee (EAMC) of Northwest A&F University (2011–31,101,684). All the operations and experimental procedures complied with the national Standards of Laboratory Animal-Guideline for ethical review of animal welfare (GB/T 35892–2018) and Guide for the Care and Use of Laboratory Animals: Eighth Edition.

### Animal sources and genomic DNA isolation

Qinchuan cattle were raised with free access to food and killed in the National Beef Cattle Improvement Center (Yangling, China). Genomic DNA was extracted from cattle blood samples, using standard method [35], and stored at −20°C until subsequent analyses.

### Real-time quantitative PCR analysis of gene expression patterns

Fifteen tissue types (testis, subcutaneous fat, liver, lung, brain, kidney, longissimus thoracis, heart, spleen, large intestine, small intestine, omasum, rumen, abomasum, and reticulum) were obtained from three adult Qinchuan cattle. Total RNA was extracted from the tissues using a Total RNA kit (Tiangen, Beijing, China), and cDNA was generated using a PrimeScript™ RT Reagent kit with gDNA Eraser (Perfect Real Time; TaKaRa, Dalian, China). Real-time quantitative PCR (qPCR) was performed using a SYBR Green PCR Master Mix kit (TaKaRa, Dalian, China), the 7500 real time PCR System and the sequence detecting software SDS V 1.4.0 (Applied Biosystems, U.S.A.). All the procedures were performed in accordance with the manufacturer’s instructions. All the primers used for qPCR are listed in Table 1. *β-actin* was included as the endogenous control gene. The relative expression levels of the target mRNAs were analyzed using the 2^−ΔΔ*C*^_t_ method [36].

**Table 1:** Primers utilized in the present study

Reaction	Name	Primer sequence (5′–3′)	Tm (°C)	Product length (bp)	Amplified region
**RT-PCR**	β-actin	F: CACCAACTGGGACGACAT	60.0	202	320–521
		R: ATACAGGGACAGCACAGC			
	SIRT4	F: GGTCCTCTGCTTGGATTGTG	60.0	131	533–663
		R: CCTCGGTGAGAAAGACATCG			
	E2F1	F: TGCTCTCGGAGGACGCTGAC	60.0	98	698–795
		R: GATCACCATGACCATCTGCTCTGC			
	CEBPβ	F: GCACAGCGACGAGTACAAGATCC	60.0	158	816–973
		R: GCGACAGTTGCTCCACCTTCTTC			
	HOXA5	F: TAGTTCCGTGAGCGAGCAATTCAG	60.0	80	140–219
		R: GATCCATGCCATTGTAGCCGTAGC			
	IRF4	F: GCTCCACATCTGCCTCTACTACCG	60.0	156	807–962
		R: TTCTTCCTCTGGCTGCTGTCCTC			
	PAX4	F: GCCGTTACTATCGCACAGGTGTC	60.0	153	542–694
		R: CTGCACAGAGCTGGCGTTGG			
	CREB1	F: CACCTGCCATCACCACTGTCAC	60.0	98	448–545
		R: AGCCAGTTGTATTGCTCCTCCTTG			
	FABP4	F: TGAGATTTCCTTCAAATTGGG	60.0	101	243–343
		R: CTTGTACCAGAGCACCTTCATC			
	CEBPα	F: ATCTGCGAACACGAGACG	60.0	73	331–403
		R: CCAGGAACTCGTCGTTGAA			
	PPARγ	F: GAGATCACAGAGTACGCCAAG	60.0	216	1146–1361
		R: GGGCTCCATAAAGTCACCAA			
	ACCα	F: CTCCAACCTCAACCACTACGG	60.0	171	3949–4119
		R: GGGGAATCACAGAAGCAGCC			
	LPL	F: ACGATTATTGCTCAGCATGG	60.0	130	2955–3084
		R: ACTTTGTACAGGCACAACCG			
**Promoter cloning**	SIRT4-PF/PR	F: *CGGGGTACC*TGGTCGCCTAAGCAGGTCAA	66.0	2121	−2077/+44
		R: *CCGCTCGAG*CCCACACTCTCACCTCCAAT			
	SIRT4-P1	*CGGGGTACC*TGGTCGCCTAAGCAGGTCAA	65.0	2121	−2077/+44
	SIRT4-P2	*CGGGGTACC*GGCATGTGATTTTTGTTGTGGC	65.0	1829	−1785/+44
	SIRT4-P3	*CGGGGTACC*CTACAGGCAGATTCTTTACCA	63.5	1589	−1545/+44
	SIRT4-P4	*CGGGGTACC*CGTGCTATCATGTCCAACTC	64.0	1284	−1240/+44
	SIRT4-P5	*CGGGGTACC*GATCCAACCAATCAACCCTA	62.0	1005	−961/+44
	SIRT4-P6	*CGGGGTACC*AACACGAGTTTTACGCAAGC	61.5	631	−587/+44
	SIRT4-P7	*CGGGGTACC*TCGTCATGGCGGGGTATTGG	62.0	446	−402/+44
	SIRT4-P8	*CGGGGTACC*TTGGGCCATCTCTTGTCGAA	61.0	204	−160/+44
	SIRT4-P9	*CGGGGTACC*GAATCGGGTTGACTTG	63.0	104	−60/+44
	SIRT4-R	*CCGCTCGAG*CCCACACTCTCACCTCCAAT			
**Site-mut and EMSA**	E2F1 forward	TATTTCAAGTCGTCAT*GGCG*GGGTATTGGGAAAAGT			−411/−376
	E2F1 reverse	ACTTTTCCCAATACCC*CGCC*ATGACGACTTGAAATA			
	mE2F1 forward	TATTTCAAGTCGTCATGGGTATTGGGAAAAGT			
	mE2F1 reverse	ACTTTTCCCAATACCCATGACGACTTGAAATA			
	CEBPβ forward	CATGGCGGGGTATTGG*GAAA*AGTTTTCAATTAGCAA			−398/−363
	CEBPβ reverse	TTGCTAATTGAAAACT*TTTC*CCAATACCCCGCCATG			
	mCEBPβ forward	CATGGCGGGGTATTGGAGTTTTCAATTAGCAA			
	mCEBPβ reverse	TTGCTAATTGAAAACTCCAATACCCCGCCATG			
	HOXA5 forward	ATTGGGAAAAGTTTTC*AATT*AGCAATAATCGCGCCT			−387/−352
	HOXA5 reverse	AGGCGCGATTATTGCT*AATT*GAAAACTTTTCCCAAT			
	mHOXA5 forward	ATTGGGAAAAGTTTTCAGCAATAATCGCGCCT			
	mHOXA5 reverse	AGGCGCGATTATTGCTGAAAACTTTTCCCAAT			
	IRF4 forward	TCGTGGATTGAATCTG*TTTC*CCTGCATACAGTTACA			−230/−195
	IRF4 reverse	TGTAACTGTATGCAGG*GAAA*CAGATTCAATCCACGA			
	mIRF4 forward	TCGTGGATTGAATCTGCCTGCATACAGTTACA			
	mIRF4 reverse	TGTAACTGTATGCAGGCAGATTCAATCCACGA			
	PAX4 forward	CGGGGAATGTTATGCA*AATT*AGCATGACGTCACAGA			−99/−64
	PAX4 reverse	TCTGTGACGTCATGCT*AATT*TGCATAACATTCCCCG			
	mPAX4 forward	CGGGGAATGTTATGCAAGCATGACGTCACAGA			
	mPAX4 reverse	TCTGTGACGTCATGCTTGCATAACATTCCCCG			
	CREB1 forward	GTTATGCAAATTAGCA*TGAC*GTCACAGAGACGAATC			−91/−56
	CREB1 reverse	GATTCGTCTCTGTGAC*GTCA*TGCTAATTTGCATAAC			
	mCREB1 forward	GTTATGCAAATTAGCAGTCACAGAGACGAATC			
	mCREB1 reverse	GATTCGTCTCTGTGACTGCTAATTTGCATAAC			
**siRNA**	siSIRT4	F: GGUACUGGGCUAGAAACUUTT			
		R: AAGUUUCUAGCCCAGUACCTT			
	siE2F1	F: CCUCUUCGACUGUGACUUUTT			
		R: AAAGUCACAGUCGAAGAGGTT			
	siCEBPβ	F: CCUCGCAGGUCAAGAGCAATT			
		R: UUGCUCUUGACCUGCGAGGTT			
	siHOXA5	F: GGAGAUCAUAGUUCCGUGATT			
		R: UCACGGAACUAUGAUCUCCTT			
	siIRF4	F: GGGCACUGUUUAAAGGGAATT			
		R: UUCCCUUUAAACAGUGCCCTT			
	siPAX4	F: GGAGCCUGAGACUCCUGAATT			
		R: UUCAGGAGUCUCAGGCUCCTT			
	siCREB1	F: GGUGCCAACUCCGAUUUAUTT			
		R: AUAAAUCGGAGUUGGCACCTT			
	si-NC	F: UUCUCCGAACGUGUCACGUTT			
		R: ACGUGACACGUUCGGAGAATT			

The restriction enzyme sites (Kpn I and Xho I) are indicated by the italicized letters in the forward (F) and reverse (R) primers used for promoter cloning and generating overexpression systems. The underlined bases are core putative TF binding sites. Abbreviation: EMSA, electrophoretic mobility shift assay.

### Immunofluorescence assays

Bovine adipocytes were fixed with 4% paraformaldehyde for 20 min at room temperature and permeabilized with 0.1% Triton X-100 for 10 min. Non-specific binding was blocked by treating permeabilized bovine adipocytes with 5% BSA for 30 min. These cells were then incubated with primary antibody against SIRT4, 2 µg/ml (ab10140; Abcam, U.K.), at 37°C for 2 h, and subsequently incubated with 2% BSA and Cy3-conjugated Donkey Anti-Rabbit IgG (Sangon Biotech, China) at 1:100 dilution for 30 min. All washes were performed using 1× PBS. An anti-fade solution containing 50 ng/ml DAPI (Solarbio, China) was also used to stain the nuclei. Finally, images of the immunofluorescence staining were captured with an Olympus IX71 microscope (Olympus Optical, Tokyo, Japan).

### Promoter cloning and generation of luciferase reporter constructs

To clone the bovine *SIRT4* promoter region, gene-specific primers (*SIRT4*-PF/PR, Table 1) were designed to amplify a 2-kb promoter region upstream of the TSS. PCR amplifications were performed using genomic DNA, from Qinchuan cattle blood, as a template and KOD DNA Polymerase (Toyobo, Osaka, Japan). After digestion with the restriction enzymes Kpn I and Xho I (TaKaRa, Dalian, China), the 2-kb bovine *SIRT4* promoter fragment, was ligated on to the pGL3-basic vector and named as pGL3-P1. Using pGL3-P1 as a template and with specific primers (Table 1) designed to incorporate Kpn I and Xho I, plasmids with unidirectional deletions in the promoter sequence, namely pGL3-P2, P3, P4, P5, P6, P7, P8, and P9, were generated by PCR amplification.

### Cell isolation, culture, and transfection

Bovine adipocytes were isolated from inguinal adipose tissue collected from 3-day-old post-natal Qinchuan fetal cattle. The inguinal adipose tissue was minced and digested at 37°C for 1.5 h with 1 mg/ml collagenase type I (Invitrogen, U.S.A.), followed by filtration through a nylon mesh (200 μm pore size). The cells were collected by centrifugation at 1500 ***g*** for 10 min and washed thrice with Dulbecco’s modified Eagle’s medium: Nutrient Mixture F-12 (DMEM/F12; GIBCO, U.S.A.) and suspended in DMEM/F12 medium supplemented with 10% FBS (PAN-Biotech, South America) and antibiotics (100 IU/ml penicillin and 100 µg/ml streptomycin). Subsequently, the cells were seeded on to 100 mm × 20 mm cell culture dishes at a density of 5 × 10^4^ cells/cm^2^ and incubated at 37°C in a humidified atmosphere with 5% CO_2_. After 12 h, fresh DMEM/F12 medium supplemented with 10% FBS and antibiotics (henceforth, this culture medium will be referred to as DMEM/F12 medium and only the other supplementations will be mentioned) was added to the adhered cells. To verify if the isolated bovine cells were actually undifferentiated adipocytes, the medium was replaced with adipogenic differentiation inducing culture medium (DMEM/F12 medium supplemented with 0.5 mM 3-isobutyl-1-methylxanthine (IBMX), 1 μM dexamethasone (DEX), and 5 μg/ml insulin) to induce differentiation and incubated for 4 days. We found that with induction both the number and the diameter of the lipid droplets increased, which confirmed that this procedure isolated undifferentiated bovine adipocytes.

Transfections were carried out in 3T3-L1 and bovine adipocytes maintained in DMEM/F-12 medium at 37°C with 100% humidity and 5% CO_2_. Cells were grown overnight in 24-well plates to a confluency of 80%. Each well was treated with the transfection mixture comprising 800 ng of the expression construct (pGL3-P1, P2, P3, P4, P5, P6, P7, P8, and P9), 10 ng of pRL-TK normalizing vector, 2 μl of X-tremeGENE HP DNA transfection reagent (Roche, U.S.A.), and 100 μl of Opti-MEM (GIBCO; Invitrogen) and incubated for 6 h (in triplicate). The pGL3-basic vector served as a negative control. Six hours after transfection, adipogenic differentiation was induced in the 3T3-L1 and bovine adipocytes using the adipogenic differentiation inducing culture medium and incubated for 42 h. Cell lysates were collected 48 h post-transfection and used to measure the relative transcriptional activity of each fragment using the Dual-Luciferase Reporter Assay System (Promega, Madison, WI, U.S.A.) according to the manufacturer’s instructions. Relative luciferase activities were determined using a NanoQuant Plate™ (TECAN, infinite M200PRO).

In order to elucidate the regulation of bovine adipocyte differentiation by *SIRT4* and its TFs, differentiation was induced in the undifferentiated bovine adipocytes after transfection with siRNA. To this end, undifferentiated adipocytes were grown in six-well plates until they reached a confluency of 90% (with DMEM/F-12 medium at 37°C with 100% humidity and 5% CO_2_). At this stage, they were transiently transfected with siRNA, 50 nM siRNA was added to each well. Adipogenic differentiation was induced, 6 h after transfection, in culture batches meant to be differentiated by replacing the DMEM/F12 medium with adipogenic differentiation inducing culture medium and incubated for 4 days before the total RNA was harvested. While, the medium of the culture batches meant to be left undifferentiated was replaced with DMEM/F-12 medium only and then incubated for 4 days before harvesting of the total RNA. si-NC served as a control group.

### Site-directed mutagenesis

To carry out site-directed mutagenesis, putative TF binding sites were first analyzed using the Genomatix suite. The identified putative TF binding sites for E2F1, CEBPβ, HOXA5, IRF4, PAX4, and CREB1 were then mutated using the corresponding primers (Table 1) and the QuickChange Site-Directed Mutagenesis Kit (Stratagene, La Jolla, CA, U.S.A.). The procedure was carried out in accordance with the manufacturer’s instructions. PCR conditions used were also in accordance with the instructions in the kit. The products of the above site-directed mutagenesis procedure were treated with Dpn I, and then amplified using XL10-Gold competent cells (Stratagene); the resulting vectors were sequenced for confirmation.

### Knockdown of *SIRT4* and TFs

siRNAs targetting *SIRT4, E2F1, CEBPβ, HOXA5, IRF4, PAX4*, and *CREB1* (Table 1) were designed and synthesized along with the control siRNA (GenePharma Co., Ltd., Shanghai, China). The si-NC served as a negative control. Bovine adipocytes cultured in six-well plates were transiently co-transfected with 50 nM of each siRNA and the corresponding pGL−402/+44 and pGL−160/+44 vector.

### Electrophoretic mobility shift assays

A LightShift Chemiluminescent EMSA Kit (Thermo Fisher Corp., Waltham, MA, U.S.A.) was used for this assay in accordance with the manufacturer’s protocol with some modifications. All DNA probes (Table 1) were synthesized (Invitrogen, Carlsbad, CA, U.S.A.) and labeled at the 5′ end with biotin. Nuclear extracts from bovine adipocytes were prepared using the Nuclear Extract Kit (Active Motif Corp., Carlsbad, CA, U.S.A.) according to the manufacturer’s protocol. Briefly, 10 μg of nuclear protein extract was incubated with 2 μl of 10× binding buffer and 1 μl of poly (dI-dC) in a 20-μl volume for 15 min on ice. Next, 200 fmol of 5′-biotin labeled probes were added and the reaction mixture was incubated at room temperature (26°C) for 20 min. For the competition assay, unlabeled probes or mutated probes were added to the reaction mixture 15 min before adding the labeled probes. For the super-shift assay, 10 μg of each of the following antibodies were added to the reaction mixture and then incubated on ice for 30 min before adding the labeled probes: anti-E2F1 (ab179445; Abcam, U.K.), anti-CEBPβ (sc-746; Santa Cruz, U.S.A.), anti-HOXA5 (sc-13199; Santa Cruz, U.S.A.), anti-IRF4 (sc-28696; Santa Cruz, U.S.A.), anti-PAX4 (ab42450; Abcam, U.K.), and anti-CREB1 (ab32515; Abcam, U.K.). Finally, the DNA–protein complexes were separated on a 6% non-denaturing polyacrylamide gel by electrophoresis, using 0.5× Tris-borate-EDTA buffer, for 1 h. Images were captured using the molecular imager ChemiDoc™ XRS+ system (Bio-Rad, Hercules, California, U.S.A.).

### Statistical analysis

Statistical calculations were performed using the Statistical Analysis System (SAS) v8.0 (SAS Institute, Cary, NC). Statistical significance was determined using one-way ANOVA test. Results for gene expression and luciferase assay were based on three independent experiments. Data are expressed as mean ± S.D.; *P*<0.05 was considered significant (* denotes significance at *P*<0.05 and ** denotes significance at *P*<0.01).

## Results

### Gene expression pattern and subcellular localization of *SIRT4* in bovine adipocytes

**Figure 1:**
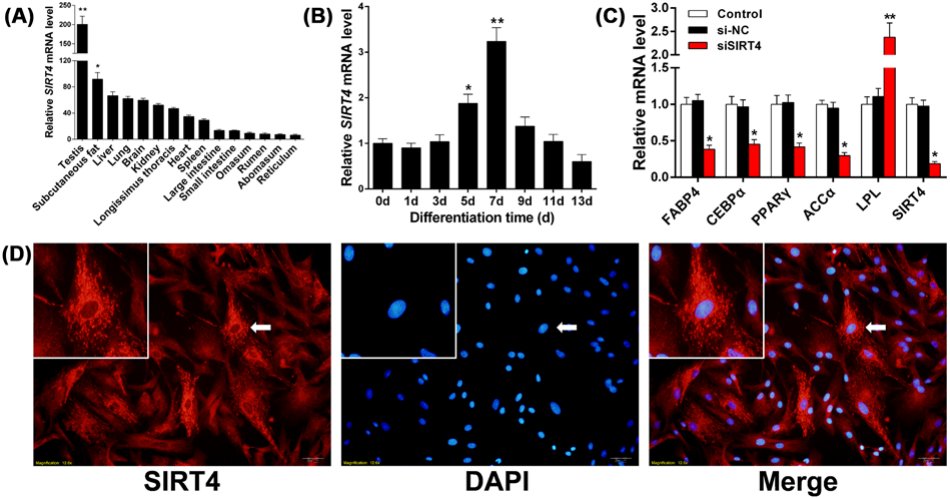
Gene expression pattern and subcellular localization of *SIRT4* (**A**) Analysis of the bovine *SIRT4* spatial expression pattern in tissues and organs. (**B**) Relative mRNA expression levels of *SIRT4* during different stages of bovine adipocyte differentiation. (**C**) During adipocyte differentiation, the expression of marker genes that promote bovine adipocyte differentiation was significantly inhibited after *SIRT4* knockdown. (**D**) SIRT4 immunofluorescence (red) in bovine adipocytes; nuclei were visualized by DAPI staining (blue). A merged figure is shown in the right panel. The top left photograph provides magnified images as indicated by the arrows. Values are represented as mean ± S.D. * indicates significance at *P*<0.05 and ** indicates significance at *P*<0.01 compared with the control group. Error bars represent the S.D.

The spatial expression pattern of bovine *SIRT4* was studied, and the results showed highest expression in the testis followed by subcutaneous fat (Figure 1A; *P*<0.05). We then measured mRNA levels of *SIRT4* during bovine adipocyte differentiation. We found that *SIRT4* expression increased before the seventh day of differentiation, which was followed by a decline over the remainder of differentiation (Figure 1B; *P*<0.05). To further explore the effect of *SIRT4* on the differentiation of bovine adipocytes, the expression levels of *SIRT4* were effectively reduced by siRNA during differentiation. Results showed that the expression of marker genes that promote bovine adipocyte differentiation was significantly inhibited after *SIRT4* knockdown (Figure 1C; *P*<0.05). The cytoplasmic subcellular localization of bovine SIRT4 was confirmed by immunofluorescence experiments; SIRT4 was found to localize in adipocyte mitochondria (Figure 1D). These results suggested that *SIRT4* might be tightly associated with adipose development.

### Isolation and sequence analysis of the core promoter of bovine *SIRT4*

**Figure 2:**
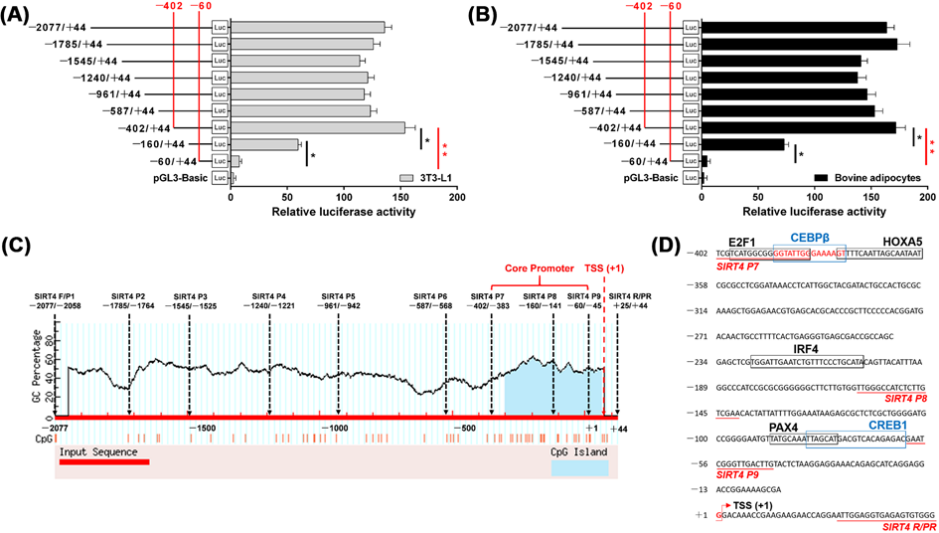
Isolation and sequence analysis of the core promoter of *SIRT4* (**A**,**B**) Depiction and reporter assay results for the series of plasmids containing 5′ unidirectional deletions in the promoter region of *SIRT4* fused in-frame to the luciferase gene. These plasmids were transfected into 3T3-L1 or bovine adipocytes. (**C**) Schematic representation of the relative loci of the potential motif binding sites in the *SIRT4* promoter. Dashed lines indicate the GC percentage as represented by the y-axis; the x-axis denotes the bp position in the 5′ UTR. (**D**) Sequence of the 5′ regulatory region of bovine *SIRT4*. Arrows mark the transcription initiation sites. The translational start site is shown in red letters. The TF binding sites are boxed and primer sequences are underlined with respective names shown below the line. * indicates significance at *P*<0.05 and ** indicates significance at *P*<0.01 compared with the control group. Error bars represent the S.D.

We further explored the activity of potential *cis*-acting elements and identified the key sequence required for their activity. We constructed a series of reporter vectors with progressively larger deletions, from the 5′ end of the promoter. Results of luciferase reporter assays using 3T3-L1 and bovine adipocytes identified a core promoter in the −402 bp/−60 bp region of *SIRT4* (Figure 2A,B; *P*<0.05). In addition, the analysis using the MethPrimer program discovered a CpG island located in the −378 bp/+18 bp region, which was found to be highly similar to the core promoter in the −402 bp/−60 bp region (Figure 2C). We further analyzed regulatory elements in the *SIRT4* −2077 bp/+44 bp promoter region using the Genomatix suite. Six motifs including TF binding sites for E2F1, CEBPβ, HOXA5, IRF4, PAX4, and CREB1 were identified in the core promoter region (Figure 2D).

### Identification of TFs that function as transcriptional activators or repressors in the *SIRT4* core promoter region

**Figure 3:**
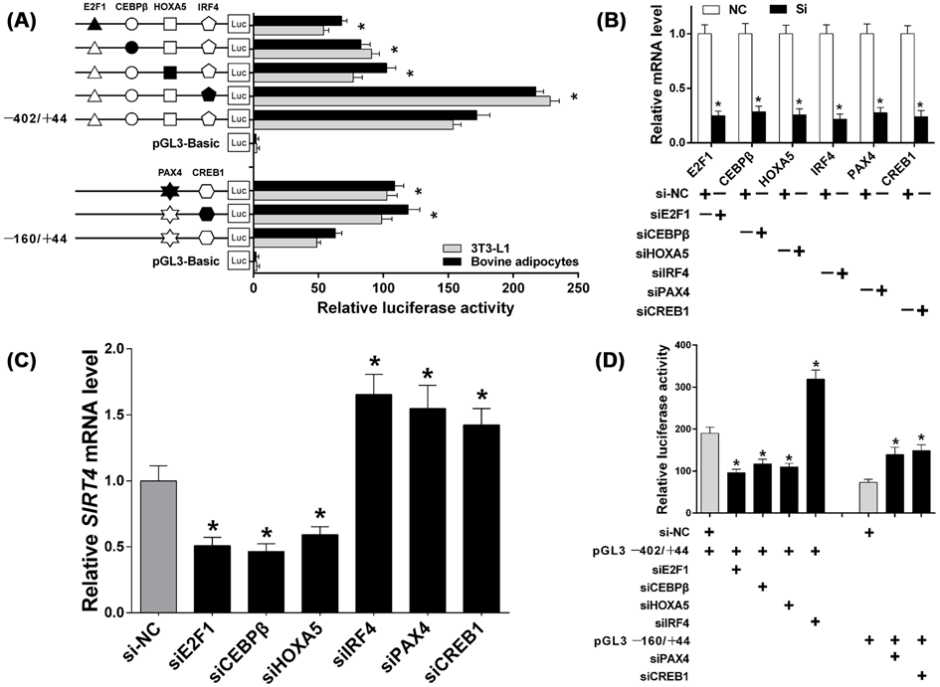
Identification of the E2F1, CEBPβ, HOXA5, IRF4, PAX4, and CREB1 as transcriptional activators or repressors in the core promoter region of *SIRT4* (**A**) Luciferase assays after transfection of constructs generated by site-directed mutagenesis of TF binding sites. White and black fill in sequence depictions represent wild and mutant types, respectively. (**B**) Knockdown efficiency of si*E2F1*, si*CEBPβ*, si*HOXA5*, si*IRF4*, si*PAX4*, and si*CREB1*. (**C**) Relative *SIRT4* mRNA expression in cells transfected with siRNAs targetting TFs. (**D**) Luciferase reporter assays after *E2F1, CEBPβ, HOXA5, IRF4, PAX4*, and *CREB1* knockdown by siRNA and co-transfection with pGL−402/+44 and pGL−160/+44 in bovine adipocytes. si-NC was used as a negative control. * indicates significance at *P*<0.05 compared with the control group. Error bars represent the S.D.

To identify the functions of TFs in the regulation of bovine *SIRT4*, we constructed a series of DNA plasmids with 4-bp point mutations in the TF binding motifs, and transfected these into 3T3-L1 and bovine adipocytes. As shown, mutating IRF4 site in the pGL−402/+44 construct and the PAX4 and CREB1 sites in the pGL−160/+44 construct resulted in a significant increase in *SIRT4* promoter activity; in contrast, mutating the E2F1, CEBPβ, and HOXA5 sites in the pGL−402/+44 construct resulted in a significant decrease in *SIRT4* promoter activity (Figure 3A; *P*<0.05). Next, siRNAs targetting *E2F1, CEBPβ, HOXA5, IRF4, PAX4*, and *CREB1* were designed, synthesized, and transfected into bovine adipocytes to detect the knockdown efficiency of respective genes. Results indicated that the expression levels of these TFs were effectively reduced (Figure 3B; *P*<0.05). We then utilized these siRNAs to determine the effect of knocking down the TFs involved in *SIRT4* expression. *E2F1, CEBPβ*, and *HOXA5* knockdown down-regulated *SIRT4* mRNA expression, whereas ablation of *IRF4, PAX4*, and *CREB1* up-regulated *SIRT4* mRNA levels (Figure 3C; *P*<0.05). To further validate the results, siRNAs targetting the TFs and pGL−402/+44 or pGL−160/+44 were co-transfected, which obviously altered activity of the *SIRT4* promoter. The transcriptional activity was significantly decreased when *E2F1, CEBPβ*, or *HOXA5* were knocked down, whereas it was increased when *IRF4, PAX4*, or *CREB1* were suppressed (Figure 3D; *P*<20.05).

### TFs bind to the core promoter region of *SIRT4*

**Figure 4:**
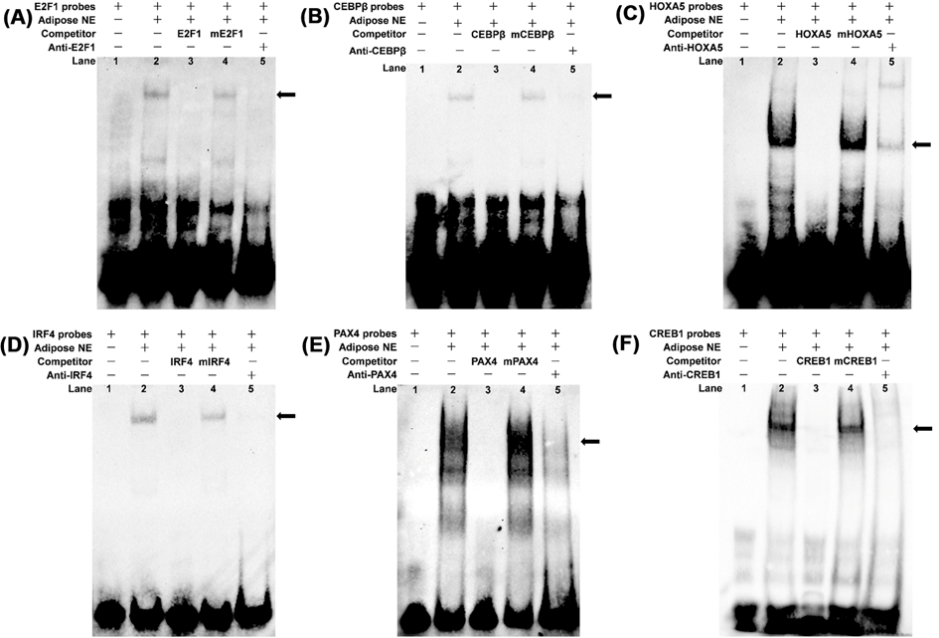
Identification of TFs binding to the core promoter of *SIRT4* using EMSAs EMSAs showing direct binding of E2F1, CEBPβ, HOXA5, IRF4, PAX4, and CREB1 to the *SIRT4* promoter *in vitro*; nuclear protein extracts were incubated with 5′-biotin labeled probes for (**A**) E2F1, (**B**) CEBPβ, (**C**) HOXA5, (**D**) IRF4, (**E**) PAX4, or (**F**) CREB1 binding sites in the presence or absence of competitor (lane 2), a 50× concentration of mutated probe (lane 3), and a 50× concentration of unlabeled probes (lane 4). The super-shift assay was conducted using 10 μg of anti-E2F1, anti-CEBPβ, anti-HOXA5, anti-IRF4, anti-PAX4, or anti-CREB1 antibodies (lane 5). The arrows indicate the main complexes. Abbreviation: adipocyte NE, adipocyte nuclear protein extract.

Electrophoretic mobility shift assays (EMSAs) were then performed to assess binding of E2F1, CEBPβ, HOXA5, IRF4, PAX4, and CREB1 to the *SIRT4* core promoter. As shown (lane 2, Figure 4A), bovine adipocyte nuclear protein binds the 5′-biotin labeled E2F1 probes and forms one predominant complex. Competition assays determined that the mutant probe had a negligible effect on this complex (lane 3, Figure 4A). At the same time, the specific E2F1/DNA interaction was prevented by competition from excess unlabeled DNA (lane 4, Figure 4A). The last lane indicates that the complex was super-shifted upon incubation with an anti-E2F1 antibody (lane 5, Figure 4A). CEBPβ, HOXA5, IRF4, PAX4, and CREB1 probes yielded similar results (Figure 4B,C,D,E, and F). In the all EMSAs, we found that the upshifted bands were diminished with antibody addition. These results indicated that the TFs bind to the core promoter region of the *SIRT4 in vitro*.

### TFs and *SIRT4* gene cooperatively regulated the relative expression of adipocyte differentiation marker genes

**Figure 5:**
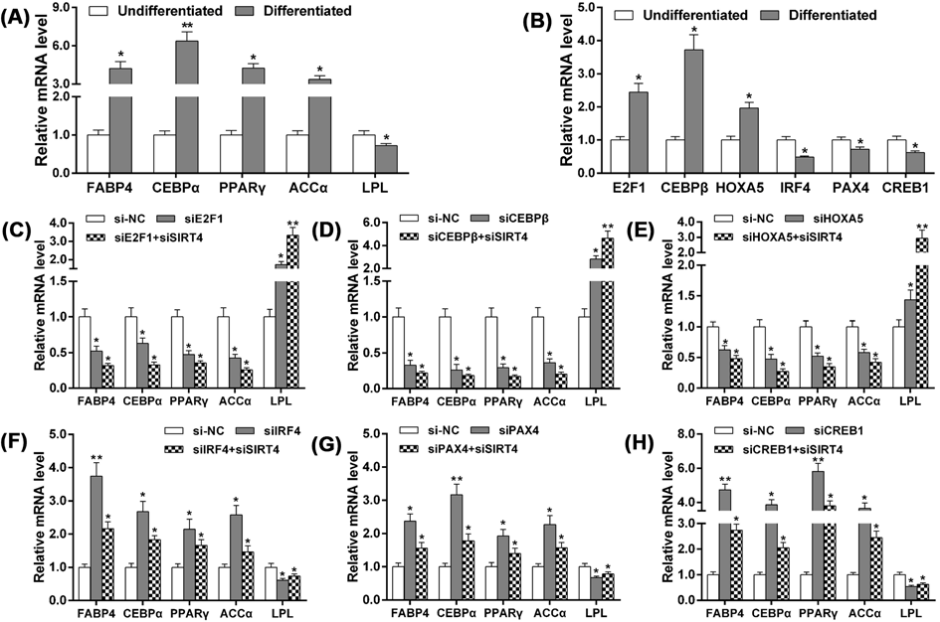
TFs and *SIRT4* cooperatively regulated the relative expression of adipocyte differentiation marker genes (**A**) The relative expression levels of marker genes in undifferentiated and differentiated stages of bovine adipocytes. (**B**) The differences in expression of TFs in undifferentiated and differentiated bovine adipocytes. (**C**–**H**) *E2F1, CEBPβ*, and *HOXA5* knockdown significantly down-regulated the expressions of marker genes that promote adipocyte differentiation; while the ablation of *IRF4, PAX4*, and *CREB1* significantly up-regulated the expressions of marker genes. *E2F1, CEBPβ, HOXA5*, and *SIRT4* had the same regulatory effect on the expression of adipogenic marker genes, and *SIRT4* coordinated with *E2F1, CEBPβ*, and *HOXA5* in promoting adipocyte differentiation. On the contrary, the regulation of *IRF4, PAX4*, and *CREB1* on the expression of adipogenic marker genes was opposite to the effect of *SIRT4*, and *SIRT4* was able to counteract the negative regulation of *IRF4, PAX4*, and *CREB1* on the adipocyte differentiation.

To confirm whether the degree to which the bovine adipocytes had differentiated was appropriate for the study, we first ascertained the relative expression levels of marker genes in undifferentiated and differentiated cultures of adipocytes; results revealed that the expression of the marker genes changed significantly in the cultures induced to differentiate (Figure 5A; *P*<0.05). The differences in expression of the six TFs were also ascertained in undifferentiated and differentiated bovine adipocytes (Figure 5B; *P*<0.05).

In order to detect the regulation of differentiation in bovine adipocytes by *SIRT4* and TFs, we knocked down the *SIRT4* and TFs using siRNAs. The results showed that these TFs have a significant regulatory effect on the expression of marker genes during adipocyte differentiation. In particular, interference of *E2F1, CEBPβ*, and *HOXA5* significantly down-regulated the expression of adipogenesis marker genes, which promote adipocyte differentiation (Figure 5C–E; *P*<0.05). However, the expression of adipogenic differentiation marker genes was significantly up-regulated with the interference of *IRF4, PAX4*, and *CREB1* (Figure 5F–H; *P*<0.05). Since these TFs bind to the core promoter region of *SIRT4*, and *SIRT4* also has a significant regulatory effect on adipocyte differentiation, we assumed that TFs coordinate with *SIRT4* to regulate adipocyte differentiation. In order to verify this hypothesis, we knocked down *SIRT4* with interference of each TF separately by siRNAs during adipocyte differentiation, and then detected the expression level of the differentiation marker genes. The results showed that TFs and *SIRT4* interact coordinately in promoting adipocyte differentiation. *E2F1, CEBPβ, HOXA5*, and *SIRT4* all increase the expression of adipogenic marker genes. Moreover, *SIRT4* synergized the effects of *E2F1, CEBPβ*, and *HOXA5* on adipocyte differentiation (Figure 5C–E; *P*<0.05). However, *IRF4, PAX4, CREB1* significantly down-regulated the mRNA expression of adipogenic marker genes, in addition, *SIRT4* antagonized the inhibitory effects of *IRF4, PAX4*, and *CREB1* on adipocyte differentiation (Figure 5F–H; *P*<0.05). Based on above results, we concluded that *SIRT4* is a vital downstream effector of these TFs in not only the pathways regulating adipocyte differentiation, but also in pathways regulating fatty acid oxidation, lipid deposition, and adipocyte growth.

## Discussion

Energy metabolism is an important aspect of metabolism; metabolism of glucose and lipid is critical to maintain cell energy homeostasis. In the physiological and biochemical processes of cells, glucose and lipid metabolism is primarily regulated by caloric restriction, oxidative stress, or other changes in energy [12]. Therefore, the focus of biological research has been on the main molecules that regulate metabolism in cell survival and homeostasis. Amongst these regulators, sirtuins have been the new research hotspots because of their important roles in regulating and maintaining glucose and lipid homeostases. Moreover, the biological functions of sirtuins have been confirmed to vary vastly from being involved in metabolism to being core regulators of cell survival [12,37].

Mammalian SIRT4 is localized to the mitochondria and plays an important role in regulating mitochondrial gene expression and fatty acid oxidation in liver and adipocytes [25,38,39]. The decreased expression level of *SIRT4* usually leads to a significant increase in the expression of enzymes involved in fatty acid oxidation metabolism [40]. *SIRT4* liver-specific KO mice display an increased expression of PPARα target genes associated with fatty acid catabolism [22]. *SIRT4* tissue-specific KO mouse models could also be critical providing a comprehensive view of SIRT4 functions in fatty acid metabolism. In the present study, we found high mRNA expression of *SIRT4* in bovine subcutaneous fat; *SIRT4* expression increased before the seventh day of differentiation and then decreased with the differentiation of bovine adipocytes; further, the expression of marker genes that promote bovine adipocyte differentiation were significantly inhibited by *SIRT4* knockdown. These results suggested that *SIRT4* might have an important role in regulating the development of bovine adipose tissue [17,18].

The E2F TF family plays a crucial role in modulation of tumor suppressor proteins and in the control of cell cycle; it has also been identified as a target of the transforming proteins of small DNA tumor viruses [41,42]. E2F1 has an additional cyclin-binding domain, and plays a key role in metabolism beyond the control of the cell cycle in non-proliferating cells [43,44]. CCAAT-enhancer binding proteins (C/EBPs) are key TFs that control cellular proliferation, differentiation, metabolism, inflammation, and numerous other responses, particularly in adipocytes [30,45]. CEBPβ are transiently induced during the early stages of adipocyte differentiation [30], and the ectopic expression of CEBPβ in 3T3-L1 promotes adipogenesis, even in the absence of adipogenic stimuli [46,47]. Expression of *Homeobox* genes is spatially and temporally regulated during embryonic development. *HOXA5* encodes a DNA-binding TF, which may regulate gene expression, morphogenesis, and differentiation [31]. *HOXA5* is controlled by DNA methylation [48], and has been shown to up-regulate the tumor suppressor p53 and AKT1 by down-regulation of PTEN [49]. *Homeobox* genes also have a functional role in determining adipose tissue expansion and body fat distribution, and it has been reported that HOXA5 promotes adipose differentiation in mice [31,50]. Interferon regulatory factors are proteins, which regulate transcription of interferons and play key roles in the JAK-STAT signaling pathway [51]. IRF4 has been implicated in acute leukemia [52], and strongly associated with pigmentation traits such as sensitivity of skin to sun exposure, freckles, blue eyes, and brown hair color [53]. IRF4 was also identified as an important regulator of adipogenesis and adipose lipid handling; it also regulates isoproterenol-stimulated glycerol release in adipose tissue both *ex vivo* and *in vivo* [54,55]. PAX family plays critical roles during fetal development and cancer growth. PAX4 is involved in pancreatic islet development, mouse studies have demonstrated PAX4 is a key mediator of the generation of insulin-producing β-cells during embryonic development [56,57]. CREB1 is a member of the leucine zipper family of DNA-binding proteins, it stimulates transcription by binding the cAMP response element, a DNA sequence present in many gene promoters [58]. CREB1 is phosphorylated by several protein kinases, and induces transcription of genes in response to hormonal stimulation of the cAMP pathway [59]. CREB1 has already been confirmed to be a well-documented TF, which is involved in adipocyte differentiation and lipid metabolism [60,61].

To elucidate the regulatory mechanism of bovine *SIRT4* expression, we analyzed the 5′ regulatory region of this gene using online prediction software, and found putative binding sites for the following TFs, namely E2F1, CEBPβ, HOXA5, IRF4, PAX4, and CREB1 at the −402/−60 bp core promoter region of the *SIRT4*. We hypothesized that these TFs might play major roles in regulating the transcriptional activity of *SIRT4*, and TFs can coordinate with *SIRT4* to regulate adipocyte differentiation. We found that E2F1, CEBPβ, and HOXA5 are transcriptional activators of *SIRT4* and IRF4, PAX4, and CREB1 transcriptional repressors of *SIRT4*. We also showed that *SIRT4* could affect the ability of these TFs in regulating the adipocyte differentiation. These results confirm the coordinate regulation by *SIRT4* and TFs on adipose differentiation.

## Conclusion

**Figure 6:**
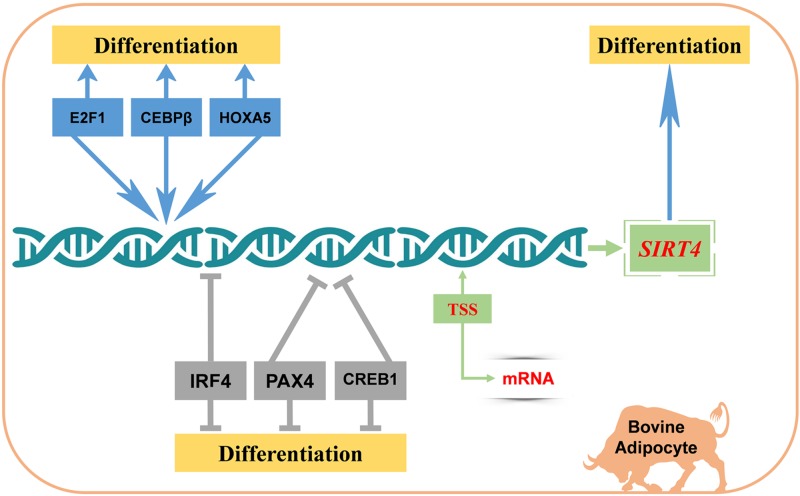
Proposed schematic summary of the regulation of differentiation of bovine adipocytes by *SIRT4* and its TFs In short, *SIRT4* promotes the differentiation of bovine adipocytes, E2F1, CEBPβ, and HOXA5 increase the *SIRT4* promoter activity, while IRF4, PAX4, and CREB1 decrease the *SIRT4* promoter activity.

In summary, we revealed high *SIRT4* expression in bovine adipose tissue, which increased before the seventh day of adipocyte differentiation. During adipocyte differentiation, the expression of marker genes that promote adipocyte differentiation was significantly inhibited after *SIRT4* knockdown. We identified the core promoter of the bovine *SIRT4* gene and determined that *SIRT4* is regulated by various TFs: E2F1, CEBPβ, and HOXA5 as transcriptional activators, whereas IRF4, PAX4, and CREB1 as transcriptional repressors. Moreover, we elucidated the regulatory mechanisms of TFs in adipocyte differentiation. Further, we verified that *SIRT4* knockdown could affect the ability of TFs in regulating the differentiation of bovine adipocytes. These data provide a foundation to better understand the transcriptional regulation and biological function of bovine *SIRT4* in adipocyte differentiation (Figure 6).

## Abbreviations

3T3-L1: mouse pre-adipose embryo fibroblast
C/EBP: CCAAT enhancer binding protein
CEBPβ: CCAAT enhancer binding protein β
CpG: 5′—C—phosphate—G—3′
CREB1: cAMP responsive element-binding protein 1
DMEM/F12: Dulbecco’s modified Eagle’s medium: nutrient mixture F-12
E2F1: E2F transcription factor 1
EMSA: electrophoretic mobility shift assay
HOXA5: homeobox A5
IDE: insulin degrading enzyme
IRF4: interferon regulatory factor 4
JAK-STAT: janus kinase-signal transducers and activators of transcription
KO: knockout
KOD: thermococcus kodakarensis
MCD: malonyl-CoA decarboxylase
PAX4: paired box 4
PPARα: peroxisome proliferator-activated receptor alpha
qPCR: real-time quantitative PCR
si-NC: negative control siRNA
SIRT4: sirtuin 4
TF: transcription factor
TSS: transcription start site
